# New Immunotherapeutic Approaches for Glioblastoma

**DOI:** 10.1155/2021/3412906

**Published:** 2021-09-13

**Authors:** Gustavo Ignacio Vázquez Cervantes, Dinora F. González Esquivel, Saúl Gómez-Manzo, Benjamín Pineda, Verónica Pérez de la Cruz

**Affiliations:** ^1^Neurochemistry and Behavior Laboratory, National Institute of Neurology and Neurosurgery “Manuel Velasco Suárez”, Mexico City 14269, Mexico; ^2^Posgrado en Ciencias Biológicas, Unidad de Posgrado, Edificio A, 1° Piso, Circuito de Posgrados, Ciudad Universitaria, Coyoacán, C.P. 04510 Distrito Federal, Mexico; ^3^Laboratorio de Bioquímica Genética, Instituto Nacional de Pediatría, Secretaría de Salud, México City 04530, Mexico; ^4^Neuroimmunology Department, National Institute of Neurology and Neurosurgery “Manuel Velasco Suárez”, Mexico City 14269, Mexico

## Abstract

Glioblastoma (GBM) is the most common primary malignant brain tumor with a high mortality rate. The current treatment consists of surgical resection, radiation, and chemotherapy; however, the median survival rate is only 12–18 months despite these alternatives, highlighting the urgent need to find new strategies. The heterogeneity of GBM makes this tumor difficult to treat, and the immunotherapies result in an attractive approach to modulate the antitumoral immune responses favoring the tumor eradication. The immunotherapies for GMB including monoclonal antibodies, checkpoint inhibitors, vaccines, and oncolytic viruses, among others, have shown favorable results alone or as a multimodal treatment. In this review, we summarize and discuss promising immunotherapies for GBM currently under preclinical investigation as well as in clinical trials.

## 1. Introduction

Glioblastoma multiforme (GBM) stands as the most frequent and aggressive form of the central nervous system (CNS) primary neoplasms. The standard protocol for treating this malignancy recommends, if possible, maximal surgical resection of the tumor mass followed by radiotherapy with adjuvant and concomitant temozolomide or a combination of procarbazine, lomustine, and vincristine (PCV schedule) for recurrent glioblastoma [1]. However, the standard of care (SOC) protocols have only prolonged from 10-12 months to 14-16 months the median of overall survival (OS) of GBM patients and only 5-year survival in 5%; thus, GBM remains an incurable disease [2].

During the early tumor formation, immune surveillance allows carrying out antitumor immune responses mediated by M1 macrophages, natural killer cells (NKs), and antigen-specific lymphocytes. However, GBM cells become able to elicit a series of mechanisms that permit the recruitment of monocytes that become tumor-associated macrophages (TAMs) as well as the recruitment of tumor tolerogenic lymphocytes like T regulatory cells (Tregs) with the production of anti-inflammatory cytokines such as TGF-*β*, VEFG, and interleukin- (IL-) 6 and 10 [3, 4], besides the expression of immune checkpoint molecules that inhibit cytotoxic immune response, cytotoxic T lymphocyte antigen-4 (CTLA-4), and programmed cell death-1 (PD-1) [5, 6]. These mechanisms contribute to the modelling of GBM microenvironment constituted of highly infiltrated TAMs and Tregs and the presence of anergic infiltrated cytotoxic lymphocytes, thus representing a barrier to the GBM treatment because of the maintenance of conditions favorable for tumor growth and immune escape [7, 8].

Considering the immunosuppressive tumor environment, new therapies are emerging focused on reactivating the immune response against tumors as promising tools for the increase in tumor clearance and the improving patient survival. Immunotherapeutic strategies encompass the use of different substances to stimulate the antitumor immune response or the elimination of immunosuppressive cells, as well as the use of substances produced by immune components to combat tumor proliferation and immune evasion. Immunotherapeutic approaches involve the passively mediated administration of monoclonal antibodies, the use of adjuvants and cytokines, or the active-mediated immunization by antigen vaccination or transplantation of activated dendritic cells or trained cytotoxic lymphocytes, exploiting the humoral and cellular components of the immune system to directly inhibit tumor growth and abrogate the tumor-mediated immune suppressive mechanisms or to activate the innate/adaptive immune response against the tumor [9]. Some of these have been successful in treating other neoplasms such as melanoma and leukemia. Here, we summarize the most recent reports of immunotherapeutic efforts against GBM.

## 2. Current Immunotherapy for Glioblastoma

**Figure 1:**
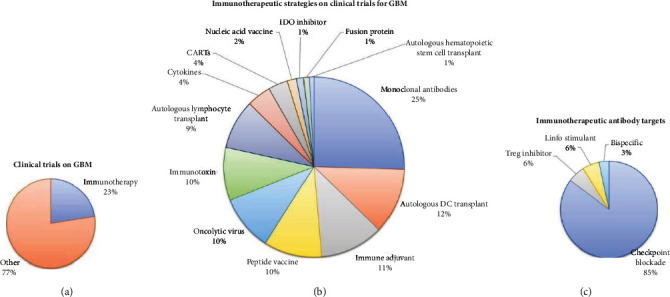
Targets of clinical trials for GBM.

Today, the development of immunotherapeutic tools to combat GBM has begun to be tested in clinical trials. Until mid-2021, 1,646 clinical trials for GBM had been registered in the clinical trial database of the United States National Institutes of Health, of which 22.53% use one or more immunotherapeutic strategies alone or in combination with SOC (Figure 1(a)).

Within immunotherapeutic strategies, monoclonal antibodies are the most frequently used agents in GBM patients (24.45% of the clinical trials; Figure 1(b)). These immunoglobulins are targeted to disrupt tumor homeostasis by promoting the activation of antitumor cytotoxic lymphocytes and inhibition of Tregs (Figure 1(c)). However, in light of the definition of immunotherapy, antibodies that block angiogenic signalling or those targeting the inhibition of growth factor receptors are not strictly considered immunotherapies due to the lack of evidence pointing to an immunogenic role of these antibodies. Among these, bevacizumab, a humanized antibody targeted against VEGF-A, is the most frequently used antibody for GBM therapy; the effectivity of bevacizumab in GBM patients has been summarized in recent reviews [10, 11], while other monoclonal antibodies tested in GBM patients are targeted against the variant III of the epidermal growth factor receptor (EGFR-vIII), the vascular endothelial growth factor receptor (VEGFR), the Hepatocyte Growth Factor Receptor (HGFR), and other receptors overexpressed on GBM malignant cells [12–25]. Furthermore, the development of bispecific antibodies with multiple targets also has been tested in GBM clinical trials.

The transplant of autologous immune cells is another strategy tested in GBM patients. This requires the previous leukapheresis of the patient blood and then separation and culturing of dendritic cells, T lymphocytes, or NK cells. These cells are stimulated and expanded *in vitro* and then transplanted back to their recipient [26]. Another variant consists of the use of autologous dendritic cells or T lymphocytes genetically transformed *in vitro* which has also recently been tested in GBM clinical trials.

The use of peptide vaccines based on malignant cell lysates or specific tumor antigens that allow the activation of antitumor immune responses represents 8% of the clinical trial tested for GBM. In some of these trials, peptide vaccination is accompanied by adjuvants that promote innate immunity; however, adjuvants have also been tried as the only immunotherapeutic resource together with SOC.

T cells armed with chimeric antigen receptors (CAR-Ts) possess an engineered surface receptor that combines the antigen-binding region of an antibody with the intracellular activation domains of T cell receptors thereby killing tumor cells by recognizing unique malignant cell surface antigens (neoantigens) [27]. CAR-Ts have evolved into a wide spectrum of molecules that have been tested against GBM. Another immunotherapeutic approach involves immunotoxins, which are engineered proteins that contain the antigen-binding regions of an immunoglobulin fused to cytotoxic molecules. The antigen-binding regions of these immunotoxins recognize tumor epitopes and selectively deliver the toxic molecules [28]. Similarly, there is the development of tumor-specific bacteria or liposomes that deliver DNA plasmids that encode proteins that interfere with tumor proliferation or encode a lymphocyte-target surface protein [29].

Oncolytic viruses are also an immunotherapeutic approach, which selectively infect malignant cells that promote cell lysis; furthermore, viral particles could activate innate immune response contributing to antitumor activity [30]. Also, recombinant cytokines such as IL-2, IL-4, IL-17, or interferon (IFN-) *α*, IFN-*β*, or IFN-*γ* have been used in GBM clinical trials. These cytokines allow the proliferation and activation of lymphocyte antitumor responses.

All these immunotherapeutic approaches are being tested in clinical trials and will be explained in detail in the next sections. Additionally, Table 1 summarizes the most relevant immunotherapies on GBM.

### 2.1. Monoclonal Antibodies for GBM Therapy

**Figure 2:**
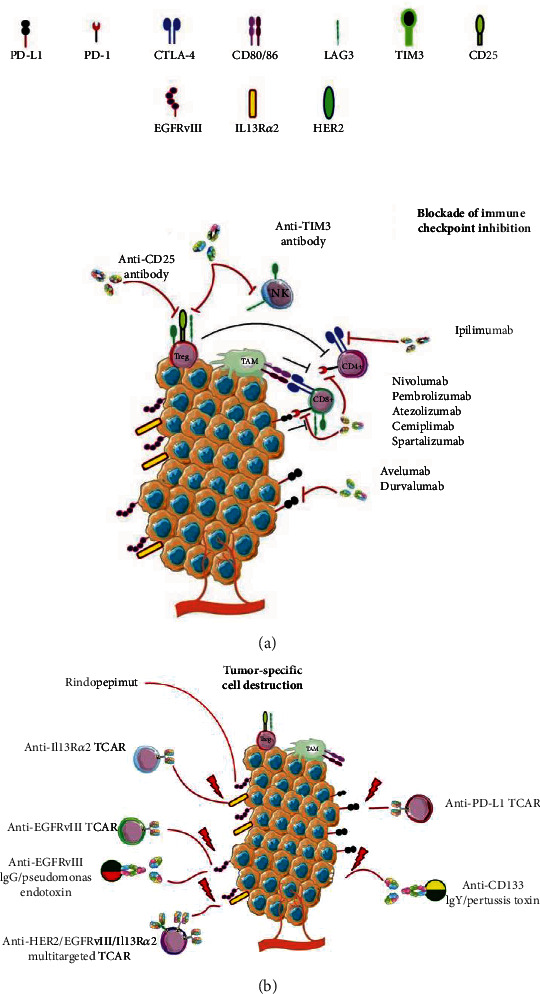
The immunotherapeutic approaches on GBM. (a) Monoclonal antibodies and their targets on the GBM microenvironment are aimed at blocking the circuit of proliferative signals in the tumor cells or restoring antitumor immune responses by blocking immune checkpoint signalling, inhibiting Treg populations, or stimulating cytotoxic lymphocytes. (b) CAR-Ts and immunotoxins designed for the recognition and elimination of malignant cells based on the expression of tumor-associated antigens present on GBM cells.

Monoclonal antibodies tested in GBM patients are targeted to block the mechanisms elicited by malignant cells that suppress antitumor immune responses (Figure 2(a)). The immune checkpoint molecules, CTLA-4 and PD-1, are cell surface proteins that negatively regulate the activation of T cells at different stages of an immune response [31]. CTLA-4 is a ligand for the costimulatory molecules B7-1/B7-2 or CD80/CD86 expressed in the cell surface of activated T lymphocytes; thus, it is a competitive inhibitor of CD28, an important costimulatory receptor for the activation of T lymphocytes [32]. The blockade of CTLA-4 allows the binding of CD80/86 with CD28 molecules expressed on the surface of T cells, activating the costimulatory signal elicited by PI3K and AKT kinases thus promoting T cell activation and proliferation [33, 34]. Furthermore, preclinical models using CTLA-4 blockade have shown to reduce the number of tumor-infiltrating Tregs, which could potentiate the antitumor response [35]. In the case of PD-1, this is a molecule expressed in mature lymphocytes; PD-1 ligation drives the blockade of activation cascades in T lymphocytes by interacting with the PD-1 ligands (PD-L1/2) that are constitutively expressed in professional antigen-presenting cells (APCs) and in a wide variety of cells in the organism [36]. Furthermore, PD-L1/2 is expressed in several tumors including GBM, where it acts as an immunosuppressive mechanism [31]; the use of antibodies against PD-1 or PD-L1/2 prevents the binding between these molecules thus avoiding phosphatase activity associated with PD-1 and allowing the TCR/CD3-mediated reactivation of exhausted T cells within the tumor [34, 37]. The development of humanized monoclonal antibodies directed against the immune checkpoint molecules, ipilimumab for CTLA-4 and nivolumab or pembrolizumab for PD-1, has shown remarkable results on OS in melanoma, lung cancer, and renal carcinoma and currently is approved for the treatment of these neoplasms [38–40]. The successful use of the immune checkpoint antibodies against melanoma brain metastasis has opened the door for the use of these agents in the treatment of GBM and its evaluation in clinical trials [41].

The combination of ipilimumab with nivolumab in untreated GBM patients has been tested in clinical trials showing partial responses accompanied by increased immune infiltrates in tumor tissue, but there was no improvement in the progression-free survival (PFS) or OS [42]. When nivolumab or ipilimumab has been used as neoadjuvant drugs, they showed that alone they had no clinical efficacy, but when they were administered before and after surgical resection, they prevented the loss of innate and adaptive immune populations induced by radiation on primary GBM [43]. Patients with recurrent GBM refractory to bevacizumab have shown greater PFS when they are treated with nivolumab [44, 45]. Besides nivolumab, pembrolizumab, the other anti-PD-1 antibody, there have been also tested patients with recurrent GBM. GMB patients who received neoadjuvant pembrolizumab with continuous adjuvant therapy after surgery showed increased CD8+ cytotoxic lymphocytes, low PD-1 levels, and then better OS and PFS results compared to GBM patients who received adjuvant, postsurgical PD-1 blockade alone [46]. Additionally, pembrolizumab has also shown prolonged PFS and OS rates after surgery on recurrent GBM patients. In this clinical trial, a slight increase in granzyme B levels was observed and no differences were found in the number of infiltrating CD4+, CD8+, or NK populations. However, a high infiltration of M0- or M2-type macrophages was described, explaining the poor antitumor immune response induced by treatment with pembrolizumab [47]. Separately, the responsiveness to these neoadjuvant treatments and the differences between lymphocyte infiltration rates into tumor tissue have been shown to be related to the mutational load on the PTEN gene. GBM patients with wild-type PTEN tumor tissue reactivity showed better outcome and increased lymphocyte infiltration than those with presented PTEN mutations [48]. On the other hand, fewer clinical studies have been reported with ipilimumab in GBM patients; patients who received ipilimumab in combination with bevacizumab showed partial radiographic responses and these drugs were well tolerated [49]. The use of ipilimumab together with temozolomide is currently being tested in clinical trials (ISRCTN84434175).

In addition to the blockade of CTLA-4 and PD-1, other checkpoint molecules such as the T cell immunoglobulin and mucin domain-containing protein 3 (TIM3) and the lymphocyte activation gene 3 (LAG-3) have been used as targets for a monoclonal antibody in GBM clinical trials. Clinical trials on the immune checkpoint inhibitors alone may have not shown high therapeutic efficacies, but the results of the immune phenotyping demonstrated that the use of these drugs allowed initiating the reactivation of antitumor immune responses. However, more information is needed focused on the combinatorial effect of these neoadjuvant treatments together with the administration of the standardized drugs against GBM.

### 2.2. Therapy Based on GBM-Associated Antigens and GBM Neoantigens

Tumor-associated antigens are molecules present in normal tissues but overexpressed in malignant cells, while neoantigens are tumor-specific molecules derived from mutations in the tumor cell genome [50]. The identification of tumor neoantigens and the identification of the abnormal expression of surface proteins in tumor cells have led to the development of T lymphocytes armed with chimeric antigen receptors (CAR-Ts) and tumor-targeted vaccines to enhance tumor-specific toxicity (Table 1).

The development and efficacy of a range of CAR-Ts targeted against different surface molecules representative of GBM tissue have been challenged in patients. IL-13 receptor *α* 2 (IL13R*α*2) is a poor patient survival prognostic indicator, which is overexpressed by more than 50% of GBM [51]. IL13R*α*2-specific CAR-Ts have been evaluated in phase I clinical trials showing the tolerability and safety of its intracranial administration. Also, it increases the necrotic volume and reduces IL3R*α*2 expression in GBM tissue compared with paired pretreatment samples as well as tumor partial remission and complete tumor remission in one of the patients enrolled in this study [51, 52]. The EGFRvIII, a mutated form of EGFR expressed in 30% of GBM [53], is related to the increase in glioma proliferation, invasion, and therapeutic resistance [54–56]. EGFRvIII has been tested for the use of CAR-Ts and to produce peptide vaccines because it is not expressed in normal brain tissue representing an ideal therapeutic target [57]. Meanwhile, EGFRvIII-targeted CAR-Ts have been shown to infiltrate and activate within tumor tissue an early response in GBM patients. However, GBM patients have shown the overexpression of immunosuppressive markers such as PD-L1 and indoleamine-2,3-dioxygenase, together with an increase in the Treg population in tumor tissue after CAR-T administration due to the primary inflammatory response elicited by the CAR-Ts [58]. Furthermore, the engineering of CAR-Ts targeting the human epidermal growth factor receptor type 2 (HER2) has also shown promising results against GBM, showing an increase in PFS and OS means in treated patients [59].

The transplant of engineered CAR-Ts has shown promising results in the treatment of GBM; however, current treatments only have focused on the administration of single-targeted cells limiting these tools by ignoring those malignant cells which do not express the target molecule of CAR-Ts.

#### 2.2.1. Immune Checkpoint Molecules as Targets for Engineered Lymphocytes

In addition to blocking antibodies against the immune checkpoint molecules, PD-1 has been recently exploited as a tool target of chimeric switch receptor-engineered lymphocytes. Cytotoxic lymphocytes armed with this receptor can recognize PD-1 expressed on the surface of tumor cells and can eliminate them due to the transmembrane and cytosolic domains of this receptor, corresponding to the activation domains of the protein CD28 [91]. A phase I clinical trial in recurrent and refractory GBM patients using these engineered lymphocytes showed the safety of the intravenous or intracranial administration of these cells, with increased levels of interferon-*γ* and IL-6, a signature of the activation of the engineered lymphocytes [92]. These results are promising; however, further clinical studies will determine the true efficacy of these cells as a treatment against GBM (Figure 2(b)).

#### 2.2.2. Preclinical Studies on Next-Generation CAR-T Cells

Multitargeted CAR-Ts for treating GBM have been started to be used in preclinical models, but evidence in clinical trials is further needed to determine their therapeutic efficacy. The development of third-generation CAR-Ts has allowed more accurate responses by modifying CAR-Ts for evasion of checkpoint inhibition as well as the expression of costimulatory molecules that allow better antitumor responses. Those have been tested against GBM, showing promising results in preclinical models. Specifically, anti-EGFRvIII CAR-Ts with a deleted expression of PD-1 have shown better antiglioma activity and longer survival in mice than in those CAR-Ts with wild-type PD-1 expression [78]. In the same line, EGFRvIII CAR-Ts expressing costimulatory molecules like CD28 or OX-40 have been shown to improve survival in an intracranial xenogenic human glioblastoma model [77]. As was mentioned above, a limitation of the use of single-targeted CAR-Ts is the elimination of just a single type of tumor cell ignoring the variability present on heterogeneous tumors such as GBM. To solve this question, the development of multiple-targeted CAR-Ts has come to be explored taking into consideration that the target should be a tumor-specific antigen to prevent off-tumor toxicities. The development of trivalent CAR-Ts specific to IL13R*α*2, the human epidermal growth factor receptor (HER-2) and EGFRvIII, was made possible by analysing interpatient variability of multiple GBM samples, showing tumor cell-specific cytotoxicity and tumor remission *in vitro* and *in vivo* models [79].

### 2.3. GBM-Associated Antigen-Targeted and Neoantigen-Targeted Drugs

The combination of different elements by genetic engineering has provided the development of several immunotherapeutic tools that have been tested against GBM. The production of immunotoxins, the antigen-binding region of immunoglobulins fused with cytotoxic agents, has shown preclinical efficacy in the treatment of GBM. The immunotoxins' utility and specificity are dictated by the targeted tumor antigen, and in recent years, many targets have been explored for GBM, mainly focused on the receptor for transferrin, transforming growth factor alpha (TGF-*α*), IL-13, IL-4, and EGFRvIII. The common toxins used to produce immunotoxins are the Pseudomonas exotoxin A and diphtheria toxin (revised review [93, 94]).

Herein, we describe the recent advances in immunotoxins against GBM in the last years. One of the targets in GBM is the cancer stem cells (CSC), which are a small population of slow-dividing and self-renewing glioma cells. The glioma stem cells express the antigen CD133, which has been related to tumor resistance to chemotherapy [95]. Recently, an IgY immunotoxin was produced against the CD133+ subpopulation of GBM CSC. This avian anti-CD133 IgY was fused with the A chain of the abrin toxin with the purpose to inactivate the ribosome. The anti-CD133-IgY-abrine showed to reduce cell viability of C6 CSC *in vitro* and decreased tumor volume after implantation of malignant glioma stem cells *in vivo*, representing a low-cost tool for the treatment of GBM [86]. Another target expressed in glioma stem cells is the Eph receptors, which are part of the receptor tyrosine kinase family. The ephrinA5 (eA5), a ligand that binds with EphA3, EphA2, and EphB2 receptors, was conjugated to Pseudomonas exotoxin A showing to kill glioblastoma cells *in vitro* [25]. The calcitonin receptor has been recently considered a target for GBM since it is expressed in a high percentage of GBM human biopsies [96]. The anti-calcitonin receptor antibody conjugated to the plant toxins dianthin-30 or gelonin showed cytotoxicity in several high-grade glioma cell lines, demonstrating that this receptor could be an effective target [97]. Other immunotoxins studied in GBM are directed to the surface proteins overexpressed in GBM cells such as EFGRvIII, VEGF, or ephrin receptors [25, 87–89], and their use in combination with other immunotherapeutic tools such as the blockade of checkpoint molecules, PD-1 or CTLA-4, demonstrated tumor clearance in immunocompetent animals [89]. Furthermore, the development of multiple-targeted immunotoxins also has shown promising results for their use against GBM [98]. As with multitargeted CAR-Ts, the use of multidirected immunotoxins represents an advantage over the single-targeted tools designed, due to their capacity to recognize a wider subset of malignant cells, while the combination of different immunotherapeutic strategies also represents an advantage because of the presence of antitumor toxins, besides the fact that the blockade of immune checkpoint inhibition provides a microenvironmental change favorable to the tumor elimination.

### 2.4. Therapeutic Vaccination against GBM

Vaccination is a type of immunotherapy focused on the tumor-associated antigens. Antigens for cancer vaccines should be expressed only by malignant cells to kill specifically tumor cells. In GBM, the EGFRvIII-targeted vaccine Rindopepimut, a selective tumor-specific antigen peptide vaccine targeting the EGFRvIII mutation, has demonstrated antitumor immune activation in GBM patients as well as longer OS than that of temozolomide-treated patients [99]. However, this vaccine showed no differences in OS compared with standard treatments in phase III clinical trials [100]. Additionally, Rindopepimut has been shown to eliminate specifically cells expressing EGFRvIII, and when it is administrated with the adjuvant granulocyte-macrophage colony-stimulating factor and treated with the standard temozolomide, dosing maintenance therapy increases median PFS and OS [101]. A recent phase II study conducted in bevacizumab-naïve patients with recurrent EGFRvIII-positive GBM showed that the addition of Rindopepimut improves OS and induced robust de novo anti-EGFRvIII antibody titers; however, additional studies should be carried out to confirm these results due the small sample size [81].

ICT-107 is another vaccine tested for GBM that consists in an autologous dendritic cell pulsed ex vivo with tumor and CSC antigens (AIM-2, HLA-A2, HER2, TRP-2, gp100, and IL13R*α*2). A clinical study using ICT-107 following conventional treatment showed a nonsignificant trend toward increased PFS. Also, the use of ICT-107 vaccine showed a reduction in CD133 expression in some GBM patients [82].

On the other hand, a preclinical study was focused on the immunogenic damage induced by radiation producing damage-associated molecular patterns (DAMPs), which activate the innate immune system and latterly the adaptive immune response. In this line, recently it was demonstrated that the administration of microvesicles released after C6 cells were irradiated reduced more than 50% tumor volume, increased the infiltrating T lymphocytes, and promoted the apoptosis of glioma cells compared with the group that was not immunized with these microvesicles [102]. The use of microvesicles derived from irradiated tumor cells could be a new approach to glioblastoma treatment.

### 2.5. Oncolytic Viruses as Activators of Tumor Elimination

In recent years, the use of oncolytic viruses has gained importance as a tool for the treatment of various solid tumors due to their tropism and selective replication in tumor cells. Evidence shows that oncolytic viruses are useful by reducing tumor bulk but also in the immune reactivation of antitumor responses [103]. The efficacy of oncolytic virus against tumors is dependent on their ability to infect and kill tumor cells specifically as well as to activate the antitumor immune response (innate and adaptive). The oncolytic viruses activate the innate immune responses through tumor-associated antigens (TAAs), damage-associated molecular patterns (DAMPs), and pathogen-associated molecular patterns produced by immunogenic cell death (PAMPs). Both DAMPs and PAMPs activate the pattern recognition receptors (PRRs) expressed in the innate immune cells, such as toll-like receptors, enhancing their phagocytic and antigen presentation activities, promoting their activation and maturation, and inducing the shift towards M1 phenotypes, which contribute to a robust antitumor immune response [104–106]. Preclinical studies have evidenced the efficacy of oncolytic viruses like measles virus, herpes simplex virus, parvovirus, adenovirus, and Zika virus against GBM [107–111]. In 2018, it was reported that a live-attenuated Zika virus vaccine killed human glioma stem cells *in vitro*, which was also related to both a reduction in the intracerebral tumor growth and prolongation of animal survival, but it was observed that this vaccine activates the antitumor immunity [112]. Recently, it was described that Zika virus increases the CD8+ T cell infiltration in the tumor microenvironment and the beneficial effects on survival and tumor volume induced by Zika virus, which are enhanced by its combination with anti-PD-1 treatment [113]. Moreover, it was described that the oncolytic type I herpes simplex virus administration in a mouse GBM model led to the infiltration of both tumor antigen-specific and viral antigen-specific CD8+ T cells, which correlated with tumor reduction [104].

In the case of clinical trials, the administration of intratumorally attenuated parvovirus resulted in a slight increase in the median OS; however, there is an increased number of infiltrating lymphocytes and IFN-*γ* levels [114]. The use of the adenoviral vector aglatimagene besadenovec has shown successful results prolonging the median OS of GBM patients to 25 months [85]. However, more clinical trials are needed to investigate the safety and efficacy of the oncolytic virus as a therapy for GBM.

### 2.6. Tryptophan Catabolism as a Target for Antitumor Immune Activation

In addition to the use of anti-PD-1/PD-L1 or anti-CTLA-4 antibodies, the search of immune inhibitory blockade mechanisms has led to the study of metabolic pathways used by malignant cells to shape tumor microenvironment and to induce immune modulation. One of these routes that become to receive attention is the catabolism of tryptophan through the kynurenine pathway (KP). Evidence shows that one of the limiting enzymes of the KP, the indoleamine-2,3-dioxygenase (IDO), is overexpressed in different neoplasms including GBM, and its expression levels correlate with increased tumor-infiltrated TAM and Treg and with patient poor prognosis [115–118]. The use of pharmacological inhibitors of IDO has shown promising results in preclinical models when combined with temozolomide or anti-PD-L1 antibodies [119–121]. In this line, it has been observed that the modulation of IDO by its inhibitor 1-methyl-l-tryptophan or knocking down IDO cells, results in a reduction of tumor growth and a longer survival period. These effects were synergistic with the use of temozolomide [120]. Recently, it was shown that dinaciclib (cyclin-dependent kinase inhibitor) downregulates KP-related genes such as IDO1, TDO2, KYAT1-4, and KMO in patient-derived GBM cell lines [122].

Additionally, L-kynurenine, the product of Trp degradation by IDO or TDO, has been involved in the GBM pathology since the kynurenine-AhR pathway can contribute to increasing the growth and motility of tumor cells and suppresses the immune response. Furthermore, the KP-derived metabolites have been implied with the generation of Tregs and the induction of apoptosis in lymphocyte subsets [123]; thus, the accumulation of these molecules could be contributing to the immunosuppressive environment within the tumor; however, the dynamics of the production of these metabolites and the expression of other KP enzymes remain less studied. In this context, our group recently found a kynurenine monooxygenase (KMO) expression and activity in human glioblastoma cell culture as well as in the GBM patient's samples. This novel finding suggests a possible KMO participation in the immunosuppressive environment since KMO is not normally expressed in astrocytes (manuscript in preparation). This information could be useful for the development of new immunotherapeutic strategies employed against GBM.

### 2.7. Success of Immunotherapies: What Does It Depend on?

As mentioned above, GBM remains as an incurable disease and the low impact of current protocols for the treatment of GBM has been associated with the high prevalence of tumor recurrence mainly due to the development of drug resistance, intratumoral heterogeneity, and the given phenotypic plasticity by CSC able to change their metabolic activity under stress conditions [124–129]. All these differences are a consequence of genetic alterations that occur in malignant cells that make GBM genetically diverse within a single tumor and between different tumors [3, 130, 131]. The genomic profiling of GBM has led to the identification of common mutations and neoantigens and to the proposal of the classification of GBM into four subtypes [3, 130, 132]. In GBM, the most common genetic alterations are the mutation of the enzyme isocitrate dehydrogenase (IDH) of which IDH^mut^ tumors present a better prognosis; with the unmethylation of the 8-methylguanosine methyltransferase (MGMT) promoter, GBM with methylated MGMT show a better prognosis than that with unmethylated MGMT and EGFR amplification [130].

In the case of the different immunotherapeutic approaches reviewed here that are on clinical trials, it is noteworthy that some of them report differences in the clinical outcomes based on genotypic and phenotypic characteristics of the patients. It was demonstrated that patients who showed a long-term follow-up after treatment with antibodies targeted to immune checkpoint molecules had methylation of the MGMT promoter, a marker of good prognosis of GBM [43, 45]. IDH^wt^ patients who showed a good response to these antibodies also showed enrichment for MAPK mutations, while nonresponsive patients showed enrichment of PTEN mutations [48].

The use of CAR-Ts and peptide vaccines against tumor-specific antigens and neoantigens has demonstrated clearance of tumor cells that carry such targets [51, 59, 81]; however, these immune strategies could act as a selective pressure by not eliminating the portion of malignant cells that do not carry the antigenic target [133]. Therefore, the ideal would be to perform more in-depth molecular screenings or the use of multiple-target strategies.

Finally, the genetic profiling of patient-derived tumors could show useful information on which therapeutic strategies would be optimal for an individual but also to monitor how different tumors respond to a certain treatment.

## 3. Conclusions

Herein, the immunotherapeutic strategies that are currently accepted for GBM treatment and those which are in the first stages of the clinical study with promising results were discussed. Until now, preclinical studies have led to the expansion of the knowledge of the immunological landscape occurring in GBM, allowing the development of more accurate tools that stimulate an antitumor response. On the other hand, the immunotherapeutic strategies tested in GBM patients have not shown the efficient results expected in terms of OS; however, evidence of antitumor immune activation and increase in tumor progression-free survival are promising traits for further development of different immunological strategies to combat GBM. Furthermore, the use of combined immunotherapy strategies to combat malignant neoplasms shows encouraging results in preclinical models that lay the foundation for the beginning of its study in clinical protocols. A combination of schemes that override tumor-induced immunosuppressive mechanisms, together with those strategies that potentiate the activation of antitumor immune response, is worthy of attention. Deeper research of the immune behavior of GBM could lead to the development of more efficient tools for the treatment of this disease and therefore to the clinical acceptance of these strategies for a generalized treatment program to combat GBM.

## Figures and Tables

**Table 1:** Current immunotherapies in GBM, their target, and their effect.

Immunotherapeutic	Target	Treatment	Effects	Adverse effects	Reference
*Monoclonal antibodies*					
Nivolumab	Antireceptor programmed cell death protein-1 (PD-1) inhibits the interaction between PD-1 and its ligands	(i) Nivolumab given 3 weeks before surgery and every two weeks after surgery (*n* = 30 patients)	Clinical (i) Median progression-free survival (PFS) was 4.1 months, and median overall survival (OS) was 7.3 months (ii) Enhances expression of chemokine transcripts such as *CXCL10*, *CCL4*, and *CCL3L1* and downregulation of *CRP*, *SSX4*, and *CR2* (iii) Enhances TCR clonal diversity among tumor-infiltrating T lymphocytes (iv) Increases immune cell infiltration	(i) 9% of patients showed liver function alterations (ii) 3% of patients experienced grade 2 hyperthyroidism and anemia (ii) 3% of patients experienced rash	[43–45]
		(i) Patients with recurrent high-grade gliomas received nivolumab every two weeks (*n* = 50 patients). 43 patients received nivolumab in conjunction with bevacizumab. 7 patients received nivolumab monotherapy. All patients received radiation and chemotherapy before immunotherapy	(i) Median PFS was 4.3 months, and median OS was 6.5 months	(i) The more common adverse events were fatigue and constipation	
		(i) Nivolumab (mg/kg) or bevacizumab (10 mg/kg) every 2 weeks (*n* = 369 patients diagnosed with recurrent GBM, 184 for nivolumab, and 185 for bevacizumab)	(i) No survival benefit was observed compared with the bevacizumab group	(i) In the nivolumab group, 8.2% of patients showed alanine aminotransferase (ALT) increase, 14.8% of patients experienced diarrhea, and 9.3% reported rash. Other adverse events reported were hyperthyroidism (2.7%), maculopapular rash (3.8%), and pneumonitis (3.3%)	
Pembrolizumab	Antireceptor programmed cell death protein-1 (PD-1) inhibits the interaction between PD-1 and its ligands	16 patients diagnosed with GBM were randomized into the neoadjuvant pembrolizumab group who received 200 mg pembrolizumab 14 days prior to surgical resection. The neoadjuvant pembrolizumab group and the adjuvant-only group received 200 mg adjuvant pembrolizumab every 3 weeks	Clinical (i) Patients receiving adjuvant treatment only showed a median OS of 228.5 days, whereas median survival in the neoadjuvant group was 417 days (ii) Neoadjuvant pembrolizumab improves PFS (iii) Increases numbers of cytotoxic CD8+ lymphocytes (iv) The neoadjuvant group demonstrated immune cell activation within the tumor microenvironment that represses the cell cycle-related transcriptional activity of tumor cells	(i) 67% of patients in the neoadjuvant group experienced grade 3-4 adverse events such as pneumonitis and ALT increase. Common events were weakness (50%), headache (47%), and hyperglycemia (37%) (ii) Nausea, dizziness, and fatigue were the most common events	[46–48]
		(i) 15 patients with recurrent GBM received pembrolizumab (2 doses before surgery and every 3 weeks afterwards)	(i) Tumor microenvironment was markedly enriched for CD68+ macrophages	(i) Patients showed 31 grade 1 and 18 grade 2 adverse events. The most common was fatigue (ii) 27% of patients experienced cerebral edema	
Atezolizumab	Antiprogrammed death ligand 1 (PD-L1) blocks the PD-L1 and PD-1 interaction	Atezolizumab (1200 mg intravenous) was administrated every 3 weeks until progression in patients with recurrent GBM (8 patients prior to chemotherapy and 8 prior to bevacizumab)	Clinical (i) Safe use in GBM patients and solid tumors	63% of patients presented events related to treatment in grades 1-3, fatigue, asthenia, diarrhea, headache, AST increase (6%), and brain edema (6%)	[60]
Avelumab	Anti-PD-L1 blocks the interaction of PD-L1 with its receptors PD-1 and B7.1 on T cells and antigen-presenting cells	Avelumab (10 mg/kg intravenous every two weeks) combined with axitinib (5 mg oral two times per day) was administrated to recurrent GBM patients	Clinical (i) Safe use in GBM patients, no clinical benefit	Dysphonia (67%), lymphopenia (50%), arterial hypertension and diarrhea (48%), fatigue (46%), and mucositis (24%) in all grades Pulmonary embolism (4% grade 4), immune-related pneumonitis (2% grade 3), and immune-related hepatitis (4% grades 2-3)	[61]
Ipilimumab	Cytotoxic T lymphocyte-associated antigen (CTLA-4)	Ipilimumab (3 mg/kg) was given in combination with nivolumab (1 mg/kg) every 3 weeks for 4 doses to GBM patients	Clinical (i) Increases tumor-infiltrated lymphocytes when it is combined with nivolumab	Fatigue (55-80%), diarrhea (30-70%), headache, dizziness, increased lipase, muscular weakness, and nausea; grade 3-4 events, increased ALT and AST, all events in combined treatment with nivolumab	[42]
LAG3 monoclonal antibody	Lymphocyte activation gene-3 (LAG3)	Mice implanted with GL-261 tumors were treated with anti-PD-1 and/or anti-LAG3 (days 7 and 10)	Preclinical (i) Increases animal survival alone or in combination with an anti-PD-1 antibody	Not reported	[62]
TIM3 monoclonal antibody	T cell immunoglobulin and mucin domain-containing 3 (TIM3)	Mice implanted with the murine cell line GL261-luc2 were treated with TIM3 alone and in combination with anti-PD-1 and stereotactic radiosurgery	Preclinical (i) Triple therapy increases animal survival compared with other arms (ii) Increases infiltration of cytotoxic lymphocytes	Not reported	[63]
Daclizumab	Interleukin-2 (IL-2) receptor (IL-2R*α*/CD25)	Patients with GBM treated with lymphodepleting temozolomide received a single dose of daclizumab (1 mg/kg) with concomitant EGFRvIII-targeted vaccination	Clinical (i) Treg depletion (ii) Stimulation of antitumor immune responses when combined with vaccination	No adverse events beyond itching, swelling, and redness at the vaccination site	[64]
Anti-GITR	Glucocorticoid-induced TNFR-related protein (GITR), a costimulatory molecule expressed constitutively on regulatory T cells and by effector T cells upon activation	Mice harboring established GL261 tumors were injected directly into the glioma core with anti-GITR	Preclinical (i) Increases animal survival (ii) Increases antitumor immune responses (iii) Treg depletion	Not reported	[65, 66]
Anti-CD137	CD137, a member of the tumor necrosis factor receptor family that has been shown to augment CD4 and CD8 T cell responses	Anti-CD137 was used in combination with tumor lysate-pulsed dendritic cells Anti-CD137 in combination with CTLA-4 blocking antibodies and focal radiation	Preclinical (i) Potentiation of dendritic cell vaccination (ii) Increases animal survival when it is combined with CTLA-4 blockade and radiation	Not reported	[67, 68]
EGFRvIII/CD3 bispecific antibody	Deletion variant III of the epidermal growth factor receptor (EGFRvIII) and CD3 on T cells	EGFRvIII/CD3 bispecific antibody was administrated in xenogeneic and syngeneic models of glioma	Preclinical (i) Redirects human T cells to generate proinflammatory and antitumor immune responses (ii) Eliminates well-established tumors *in vivo* (iii) Increases survival	Not reported	[69–71]
Ang-2/VEGF bispecific antibody	Vascular endothelial growth factor (VEGF) and angiopoietins (Ang). Ang plays a role in mediating resistance to anti-VEGF therapy in GMB	Mice bearing orthotopic syngeneic (Gl261) GBMs or human (MGG8) GBM xenografts were treated with an Ang-2/VEGF bispecific antibody	Preclinical (i) Delays tumor growth (ii) Enhances survival (iii) Reduces tumor burden (iv) Reprograms tumor-associated macrophages toward an antitumor phenotype	Not reported	[72]
Anti-PD-1/PD-L1 axis	Programmed cell death protein 1 (PD-1) and/or programmed cell death ligand-1 (PD-L1), which modulated the antitumor immunity	Retrospective series of 66 GBM patients who were treated with PD-1 inhibitors	Clinical (i) Safe and limited efficacy (ii) Improves overall survival in a small subset of patients	Nausea, dizziness, and fatigue were the most common events	[48, 73, 74]
		Anti-PD-1 alone and in combination with temozolomide was evaluated in an orthotopic murine GBM model	Preclinical (i) Combination with localized radiation therapy increases survival in mice with orthotopic brain tumors (ii) Decreases tumor growth and has a synergic effect in combination with temozolomide (iii) Increases the number of tumor-infiltrating lymphocytes (iv) Decreases frequency of Treg cells		

*CAR-T cells*					
Anti-IL13R*α*2	Tumor-associated antigen interleukin-13 receptor alpha 2	CAR-T cells were infused at a maximum dose of 10^8^ in three patients with recurrent GBM A patient with recurrent multifocal GBM received infusion of six cycles of CAR-T treatment	Clinical (i) Improves the quality of life (ii) Reduces IL13R*α*2 expression on tumor tissue (iii) After CAR-T cell treatment, regression of all tumors was found as well as increases in cytokine levels and immune cell in the cerebrospinal fluid	Adverse events attributed to T cell administration were headache, shuffling gait, and tongue deviation Infusion of CAR-T cells was not associated with any toxic effects of grade 3 or higher	[51, 52, 75]
		CAR-T cells (0.2-2 × 10^6^ cells) were intratumorally infused in the orthotopic human GMB models with patient-derived tumor sphere lines	Preclinical (i) Improves antitumor activity and T cell persistence (ii) Increases survival (iii) Reduces tumor growth	No toxicity was observed	
Anti-HER2	Human epidermal growth factor receptor 2	Patients with progressive HER2-positive GBM (*n* = 17) received 1 or more intravenous infusions of autologous HER2-CAR VSTs (1 × 10^6^/m^2^ to 1 × 10^8^/m^2^).	Clinical (1) Safe and clinical benefit for 8 of 17 patients	No dose-limiting toxic effects were observed	[59, 76]
		Anti-HER2 CAR-T cells alone or in combination with anti-PD-1 were evaluated in U251 cells	Preclinical (1) Shows a stronger cytotoxic effect against U251 cells and in combination with anti-PD-1 enhancing the efficacy	Adverse events within the first 6 weeks after CAR-T infusion were headache and seizure	
Anti-EGFRvIII/CD28/OX40	Deletion variant III of the epidermal growth factor receptor (EGFRvIII) chimeric antigen receptor (CAT) expressing intracellular costimulatory domains of CD28 and OX40. CD28 enhances CAR-T cell function, and OX40 enhances activation and persistence of both CD4 and CD8 T cells	2 *μ*L of CAR-T cells were intratumorally injected in an orthotopic human glioma xenograft model	Preclinical (i) Cytolytic potential against U87 cells (ii) Enhances antitumor activity (iii) Increases mean survival	No adverse events were observed	[77]
PD-1^KD^ EGFRvIII-CAR-T	Deletion variant III of the epidermal growth factor receptor (EGFRvIII) chimeric antigen receptor with PD-1 blockade	For *in vitro* experiments, PD-L1^WT^EGFRvIII^+^ U373 cells or PD-L1^KO^EGFRvIII^+^ U373 cells were exposed to EGFRvIII-CAR-T/PD-L1^KD^. For the glioma xenograft mouse model, the CAR-T cells were administrated on day 10 after U373-eGFP cells were injected	Preclinical (i) Enhances the lytic activity of EGFvIII-CAR-T cells against PD-L1^+^ EGFRvIII^+^ GBM cells (ii) Higher antiglioma activity (iii) Increases survival	Not reported	[78]
HER2/IL13R*α*2/EphA2	Human epidermal growth factor receptor (HER2), interleukin-13 receptor subunit alpha-2 (IL13R*α*2), and ephin-A2	CAR-T cells (1 × 10^6^ T cells) were injected intratumorally on days 5 and 12 following tumor injection in nonobese diabetic severe combined immunodeficient mice implanted with patient-derived GBM cell lines	Preclinical (i) Kills 100% of tumor cells (ii) Enhances antiglioma activity (iii) Increases survival	Not reported	[79]

*Peptide vaccine*					
Rindopepimut vaccine	Peptide vaccine against EGFRvIII	Rindopepimut was administrated to bevacizumab-naïve patients with recurrent EGFRvIII-positive GBM	Clinical (i) Improves progression-free survival (small population) (ii) Reduces corticosteroid use (iii) Induces de novo anti-EGFRvIII antibody titers (iv) No differences in overall survival compared to standard of care (phase III study)	The adverse events related to Rindopepimut were primarily erythema and pruritus, brain edema, hypersensitivity reaction, and fatigue	[80, 81]
			Preclinical (i) EGFRvIII-specific antibody derivate from immunized patients' serum killed EGFRvIII-positive tumor cells		
ICT-107 vaccine	Autologous vaccine consisting of patient dendritic cells pulsed with class I peptides from tumor-associated antigens (six synthetic class I peptides from antigen isolated from immunoselected melanoma-2 (AIM-2), melanoma-associated antigen-1 (MAGE1), tyrosinase-related protein-2 (TRP-2), glycoprotein 100 (gp100), human EGFR-2 (HER2/neu), and IL13R*α*2)	Vaccine was administered intradermally every 2 weeks for 3 consecutive doses in GBM patients	Clinical (i) Well tolerated (ii) Expression of AIM2 and MAGE1 antigens in the prevaccine tumors correlated with prolonged survival in newly diagnosed glioblastoma patients (iii) Increases progression-free survival in the intent-to-treat population with the maintenance of life quality in glioblastoma patient's HLA-A2+	Adverse events were diarrhea, fatigue, flushing, pruritus, rash, redness on skin, and vomiting	[82, 83]
		Vaccine was administrated weekly ×4 followed by 12 months of adjuvant temozolomide in newly diagnosed patients with GBM		The most frequent adverse events were fatigue, convulsions, and nausea	

*Virus-delivered toxin*					
Aglatimagene besadenovec (AdV-tk)	Adenoviral vector expressing the herpes simplex virus thymidine kinase gene followed by antiherpetic prodrug.	AdV-tk (1 × 10^11^ and 3 × 10^11^ vector particles, *n* = 3 and 5, respectively) was injected into the tumor bed in pediatric patients with malignant glioma. After surgery, valacyclovir was given by 14 days and radiation started within 8 days of surgery AdV-kt plus valacyclovir in combination with the current standard care (surgical resection followed by radiation and temozolomide) was given to 48 patients diagnosed with malignant glioma	Clinical (i) Well tolerated (ii) Improves median overall survival (8.9 months for dose level 1 and 25.3 months for dose level 2) (iii) AdV-kt+valacyclovir+current standard care improves median overall survival compared with standard care alone (17.1 months vs. 13.5 months)	The adverse events were fever, fatigue, and nausea/vomiting No dose-limiting toxicities were found Common immunotherapy-related symptoms were fever, fatigue, and headache	[84, 85]

*Antibody-delivered toxins*					
Anti-CD133 IgY-abrin	CD133, a cancer stem cell marker in glioblastoma.	Nude mice implanted with malignant glioma stem cells were administrated with a single dose of immunotoxin (1.34 *μ*g/kg) by an intratumoral route or 1.34 *μ*g/kg every week for 3 weeks by an intraperitoneal route	Preclinical (i) Reduced tumoral cell viability *in vitro* (ii) Decreases tumor volume	Not reported	[86]
eA5 ligand-Pseudomonas exotoxin	EphA2, EphA3, and EphB2	U251 MG cells treated with the cytotoxin (1 *μ*g/mL; IC50 of at least 10^−11^)	Preclinical (i) Neutral to normal cells (ii) Effectively killed tumor cells	Not reported	[25]
Anti-VEGF-Pseudomonas exotoxin	Vascular endothelial growth factor (VEGF)	The plasmid was locally administrated in a murine malignant glioma model	Preclinical (i) Inhibition of angiogenesis (ii) Decreases tumor volume	Not reported	[87]
D2C7-Pseudomonas exotoxin	Epidermal growth factor receptor (EGFR) and deletion variant III of epidermal growth factor receptor (EGFRvIII)	Orthotopic tumor models (43, NR6M, D270MG) expressing EGFRwt or EGFRvIII were administrated with D2C7-exotoxin (1 mg by ALZET osmotic minipumps)	Preclinical (i) Prolongs survival (ii) D2-C7 monotherapy induces T cell-mediated antitumor immune response and increases 27% of median survival (iii) D2C7 in combination with *α*-CTLA-4 increases 80% median survival, and that in combination with *α*PD-1 increases 120% median survival	No adverse effects were observed after intracerebral administration of D2C7	[88–90]
		Murine glioma model (CT-2A-dmEGFRvIII-Luc) was infused with D2C7-immunotoxin in combination with *α*-CTLA-4 or *α*PD-1		No toxicity-associated mortality was found	
